# Arthroscopic‐Assisted Mosaicplasty for the Repair of International Cartilage Repair Society (ICRS) Grading System Grade 4 Osteochondral Defect: A Case Report of Two Patients

**DOI:** 10.1155/cro/1667776

**Published:** 2026-04-06

**Authors:** Andri Maruli Tua Lubis, Garry Soloan, Pandya Praharsa, Refky Juliandri, Adisa Yusuf Reksoprodjo

**Affiliations:** ^1^ Department of Orthopedic and Traumatology, Dr. Cipto Mangunkusumo General Hospital, Faculty of Medicine, University of Indonesia, Central Jakarta, Jakarta, Indonesia, ui.ac.id; ^2^ Faculty of Medicine, University of Indonesia, Central Jakarta, Jakarta, Indonesia, ui.ac.id

**Keywords:** femoral condyle, knee arthroscopy, mosaicplasty, osteochondral autograft transplantation, osteochondral defect

## Abstract

**Introduction:**

Osteochondral defects (OCDs) of the knee are rare lesions that cause pain and significant morbidity. These lesions are known to have no capacity for repair and increase the risk of secondary osteoarthritis. Mosaicplasty has been demonstrated to be a feasible choice for treating OCD of the knee, with the potential to relieve knee pain and facilitate return to sports. This report evaluated functional outcomes of the knee following arthroscopic mosaicplasty for the reconstruction of OCD.

**Method:**

Two patients with OCD underwent reconstruction with arthroscopic mosaicplasty. Donor autograft was harvested from the nonweight‐bearing area of the ipsilateral knee and then implanted into the defect site. Preoperative and postoperative knee function was assessed using Tegner–Lysholm and IKDC scores.

**Result:**

For Cases 1 and 2, mosaicplasty improved the Tegner–Lysholm score by 23 and 74 points and increased the IKDC score by 11.5% and 64.4%, respectively. No postoperative complications were recorded.

**Discussion:**

The use of arthroscopic mosaicplasty is an efficient, single‐step procedure with minimal adverse events and quick recovery. Here, we demonstrated that mosaicplasty allows for immediate structural reconstruction of the defect site, superior biomechanical properties through direct hyaline cartilage, and subchondral bone implantation in extensive, high‐grade lesions according to the International Cartilage Repair Society (ICRS) grading system classification for OCDs. The functional outcome 1 year following the procedure reveals excellent knee function and allows a patient to return to sports.

**Conclusion:**

This case series highlights current challenges in the treatment of OCD and the availability of surgical options for its treatment. Our experience demonstrates that arthroscopic mosaicplasty produced excellent postoperative functional outcomes for the treatment of OCD, allowed for return to sports, and resulted in no significant complications.

## 1. Introduction

The knee cartilage, primarily hyaline, provides a lubricated, low‐friction surface that facilitates smooth joint articulation, distributes mechanical load, and reduces stress. It consists of a dense extracellular matrix (ECM) rich in collagen, water, and proteoglycans, populated by chondrocytes derived from mesenchymal progenitor cells during development, ensuring structural integrity and biomechanical properties [1].

Osteochondral defect (OCD) is characterized by cartilage damage with or without subchondral bone involvement, found in 34%–62% of knee arthroscopies, predominantly in patients under 40, highlighting its high prevalence and clinical burden. Recent data surrounding the incidence of OCD ranges between 2.3 and 31.6 cases per 100,000 individuals, with data regarding the incidence of defects induced by trauma remaining scarce [2]. OCD often presents with pain and increases the risk of early‐onset osteoarthritis due to biomechanical disruptions, leading to excessive and altered stress distribution on surrounding cartilage and bone, accelerating degeneration. Additionally, the defect induces a proinflammatory state, activating catabolic pathways that degrade cartilage and inhibit ECM and chondrocyte synthesis. Combined with the cartilage′s avascular nature, these factors impair regenerative potential [3, 4].

A comprehensive assessment of knee function among patients with OCD is pivotal in both clinical and research contexts. Among post‐knee articular cartilage transplantation patients, the International Knee Documentation Committee (IKDC) score has been documented to be the most commonly utilized patient‐related outcome measure (PROM), and demonstrates the greatest effect size and responsiveness when compared with other PROMs [5]. The IKDC score evaluates three domains: (1) symptoms (e.g., stiffness, pain, swelling, locking sensation, and giving way); (2) sports and daily activities; and (3) current and preinjury knee function. The Tegner–Lysholm knee scores have been demonstrated to be useful in evaluating the degree of return to activity to sports and performance [6]. This Indonesian version of this questionnaire has demonstrated adequate and statistically significant correlation with SF‐36 questionnaires that evaluate physical function and pain levels, and is deemed to have acceptable validity, reliability, and responsiveness among Indonesian patients that underwent arthroscopic procedures of the knee [7].

Treatment choice depends on defect size, patient factors, and durability. The commonly applied grading system for OCD is the International Cartilage Repair Society (ICRS) arthroscopic grading system, which divides OCD into four grades (Table 1) [8]. Bone marrow stimulation (microfracture, subchondral drilling) is the first‐line for small lesions (< 2–4 cm^2^) but results in fibrocartilage, which lacks durability. Mosaicplasty osteochondral autograft transfer system (OATS) is preferred for moderate‐sized defects (1–4 cm^2^) as it restores hyaline cartilage, ensures better long‐term outcomes, and is a single‐stage procedure. It provides immediate structural integrity, allowing quicker rehabilitation, and is cost‐effective with high long‐term success. Unlike cell‐based techniques (ACI/MACI), which rely on cell differentiation, mosaicplasty directly transplants fully developed hyaline cartilage and subchondral bone, ensuring better biomechanical properties. Osteochondral allograft (OCA) transplantation is an option for larger defects (> 4 cm^2^) but carries risks of immune rejection and graft availability [9, 10].

**Table 1:** The International Cartilage Repair Society (ICRS) cartilage lesion classification method.

ICRS grade	Description
Grade 0	Normal
Grade 1	Superficial lesions (soft indentation and/or superficial fissures and cracks)
Grade 2	Cartilage defects involving < 50% of cartilage depth
Grade 3	● Cartilage defects involving > 50% of cartilage depth ● Cartilage defects involving calcified layer, but does not involve subchondral bone ● Blisters
Grade 4	Cartilage defects involving subchondral bone

Despite its advantages, mosaicplasty has limitations, including graft‐size mismatch, fibrocartilage formation between plugs, limited donor tissue, and donor‐site morbidity, especially with extensive harvesting. A 2–4‐week nonweight‐bearing period is also required [11]. However, it remains the only single‐stage and cost‐effective surgical technique that immediately restores the defect with live, organized hyaline cartilage and subchondral bone, unlike microfracture and ACI, which rely on regeneration and may produce fibrocartilage [9, 10]. This case series presents two OCD patients treated with mosaicplasty, reinforcing its role as a definitive treatment option.

## 2. Case Presentation

We present two cases of OCDs treated with arthroscopic mosaicplasty between December 2022 and October 2024, encompassing the timeline from diagnosis, surgical intervention, and postoperative follow‐up for each patient.

### 2.1. Case 1

A 17‐year‐old male presented to the orthopedic outpatient clinic, with a chief complaint of pain in the right knee that had persisted for 1.5 years. Prior to the complaint, the patient reported that he had a sports accident where he landed in an unnatural position on his right knee during a volleyball match, initially untreated. Eighteen months later, the patient presented to our clinic for persistent pain that was unrelieved by analgesics and physiotherapy.

**Figure 2:**
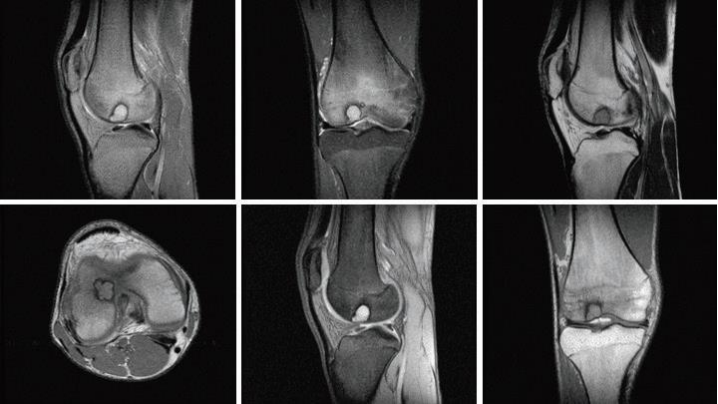
Preoperative T1‐ and T2‐weighted MRI of the right knee revealing bone contusion within the lateral femoral condyle, without any evidence for displacement of fragment or collapse.

Physical examination of the patient revealed antalgic gait and tenderness on the medial aspect of the right knee, VAS 3–4. Evaluation of right knee movement demonstrated range of motion (ROM) of 0°–150°. Knee x‐ray examinations revealed thickening of the soft tissue within the popliteal region and thickening of the suprapatellar recess, suggestive of effusion within the right knee joint, along with irregular‐shaped lucency located within the lateral femoral condyle of the right knee, suggestive of osteochondral lesions (Figure 1a,b). MRI examination found an abnormal signal intensity within the lateral femoral condyle (Figure 2). The patient was then diagnosed with OCD of the right lateral femoral condyle.

Figure 1(a) Lateral view of the right knee on plain film. Lucency is less clearly seen. (b) Anteroposterior view of the right knee on plain film where the lucency (red arrow) is more clearly seen on the lateral femoral condyle.

**Figure:**
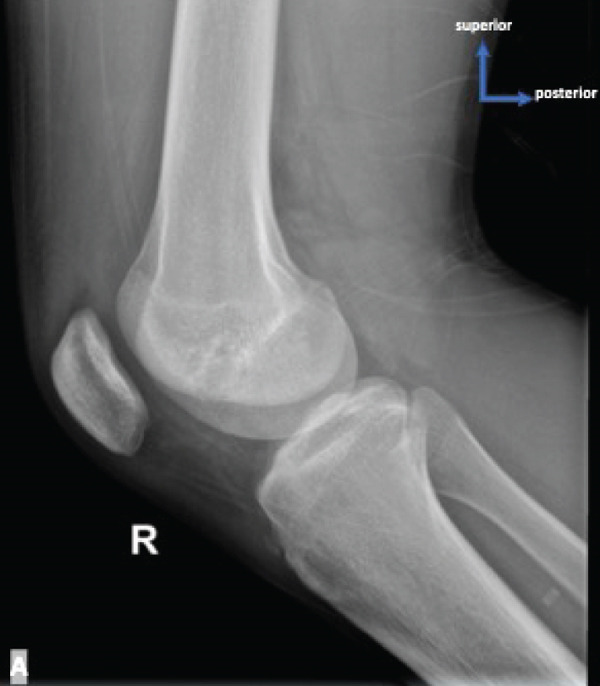
(a)

**Figure:**
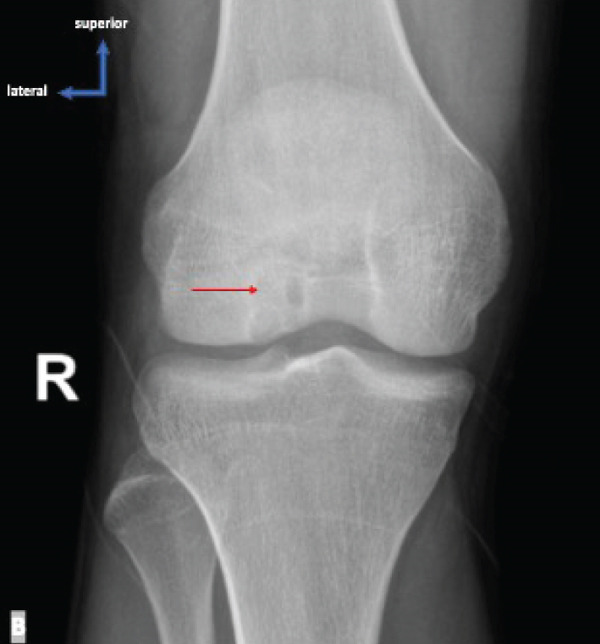
(b)

### 2.2. Case 2

A 28‐year‐old female patient presents with a complaint of left knee pain (numeric rating scale of 4–5) since 5 months ago, exacerbated by movement. Consequently, the patient experiences difficulty when walking and appears to limp. The patient has a history of a fall when walking down a stairway approximately 2 years ago. The patient then seeks traditional treatment; however, reports persistence of pain. Physical examination revealed tenderness of the left knee with VAS 5–6, a ROM of 0°–135° of knee flexion‐extension, and a dodging limp on walking. Preoperative knee x‐ray of the patient excluded fractures; however, revealed a lucency within the medial femoral condyle of the left knee suggesting a defect on the cartilage and its underlying subchondral bone (Figure 3a,b). MRI concluded that there is an OCD on the anterior medial condyle of the left knee (Figure 4). Macroscopic evaluation through diagnostic arthroscopy revealed a defect with a diameter of 18 mm, involving the subchondral bone, and the patient was diagnosed with ICRS Grade 4 OCD.

Figure 3X‐ray of the left knee in (a) anteroposterior and (b) lateral views. The x‐ray shows no signs of fracture; however, it reveals suspicion of osteochondral defect due to the appearance of lucency within the medial femoral condyle of the left knee (red arrow).

**Figure:**
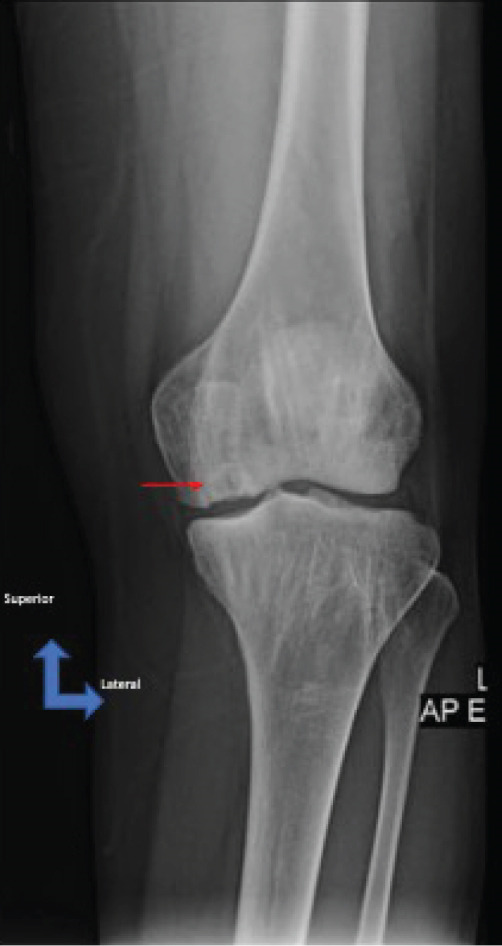
(a)

**Figure:**
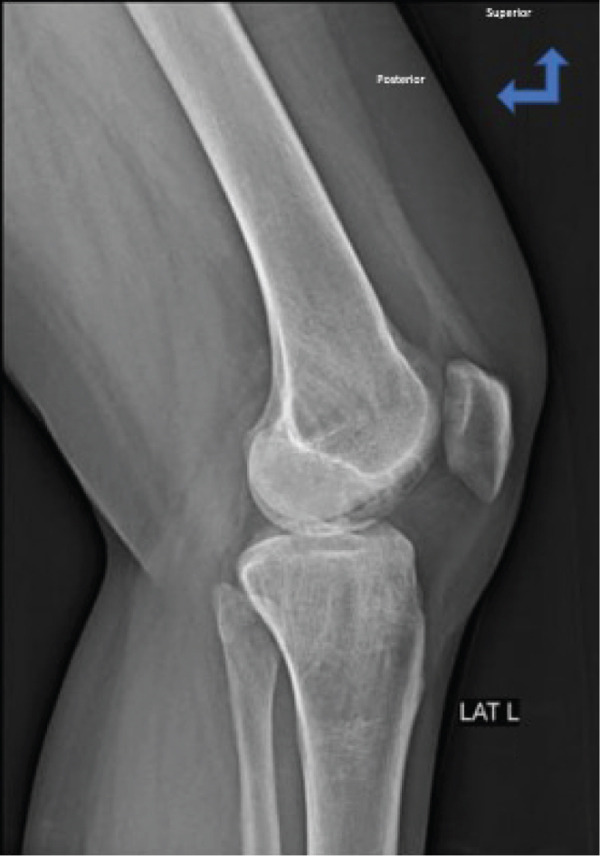
(b)

Figure 4MRI of the left knee in (a) sagittal, (b) coronal, and (c) axial views. The MRI reveals an osteochondral defect (OCD) lesion on the anterior medial condyle, accompanied by multiple subchondral cysts, with the largest measuring 0.5 cm in diameter and adjacent bone edema. Additionally, the MRI shows a normally aligned knee without evidence of dislocation or subluxation.

**Figure:**
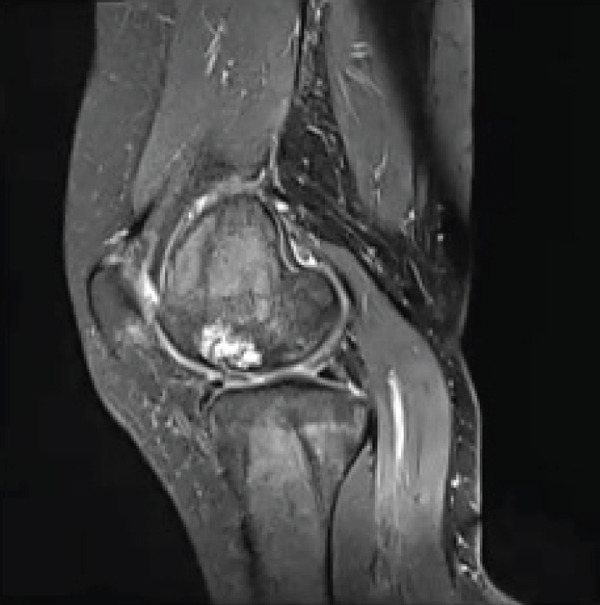
(a)

**Figure:**
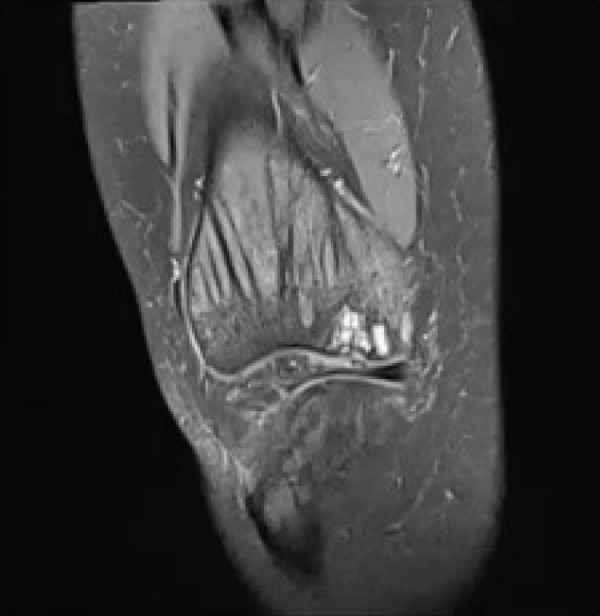
(b)

**Figure:**
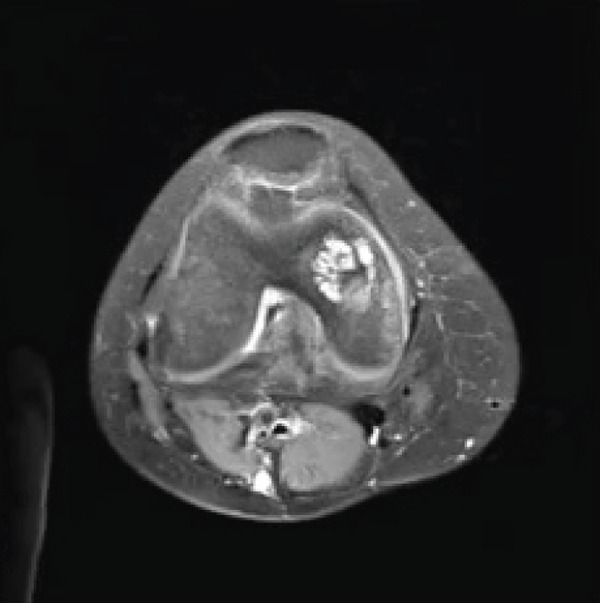
(c)

Several differential diagnoses to be considered include acute osteochondral fracture, avascular necrosis, or even degenerative conditions, such as a bone collapse due to a large subchondral cyst seen in osteoarthritis. The diagnosis of OCD in both cases is likely to occur from the history of knee trauma experienced by both patients.

Based on the findings from the physical examination and imaging studies, both patients received OATS as the surgical treatment to repair the defect. Surgery was performed with the patient under general anesthesia in supine position, with a pneumatic tourniquet applied to the root of the limb. Two grams of IV cefazolin was administered as prophylactic antibiotic. Both patients were assessed as low risk for the development of deep venous thrombosis (DVT), and therefore, no pharmacologic prophylaxis was administered. Stab incision was made to introduce the anterolateral portal, and the suprapatellar approach was used to carry out the procedure. Diagnostic arthroscopy of the first case revealed an intact anterior cruciate ligament (ACL), posterior cruciate ligament (PCL), medial meniscus, lateral meniscus, and medial and lateral gutter. We identified a round OCD on the articular surface of the lateral femoral condyle, involving the underlying subchondral bone, confirming the diagnosis of an ICRS Grade 4 OCD. (Figures 5a, 5b, and 5c **)** The defect region was stabilized by curettage and shaving to remove flapping cartilage. Defect site measurement was carried out and revealed a diameter of 6 mm. The donor osteochondral cylindrical plug measuring 6 mm, with a depth of 20 mm, was harvested from the nonweight‐bearing portion of proximal lateral femoral condyle of the ipsilateral knee using the MOSAICPLASTY system (NeoCart Osteochondral Transfer System). Following graft implantation, recipient‐site management involves ensuring that the articular surface of the donor plugs are level with the surrounding native cartilage, and the knee is evaluated by passive flexion and extension, confirming graft stability and congruity. Donor‐site defects were allowed to heal by secondary intention.

Figure 5Mosaicplasty procedure of the first case. (a) Identification and measurement of the 6‐mm osteochondral defect within the lateral femoral condyle. (b) Harvesting of osteochondral autograft from the lateral aspect of the femoral condyle. (c) Implantation of autograft into donor site and final construct.

**Figure:**
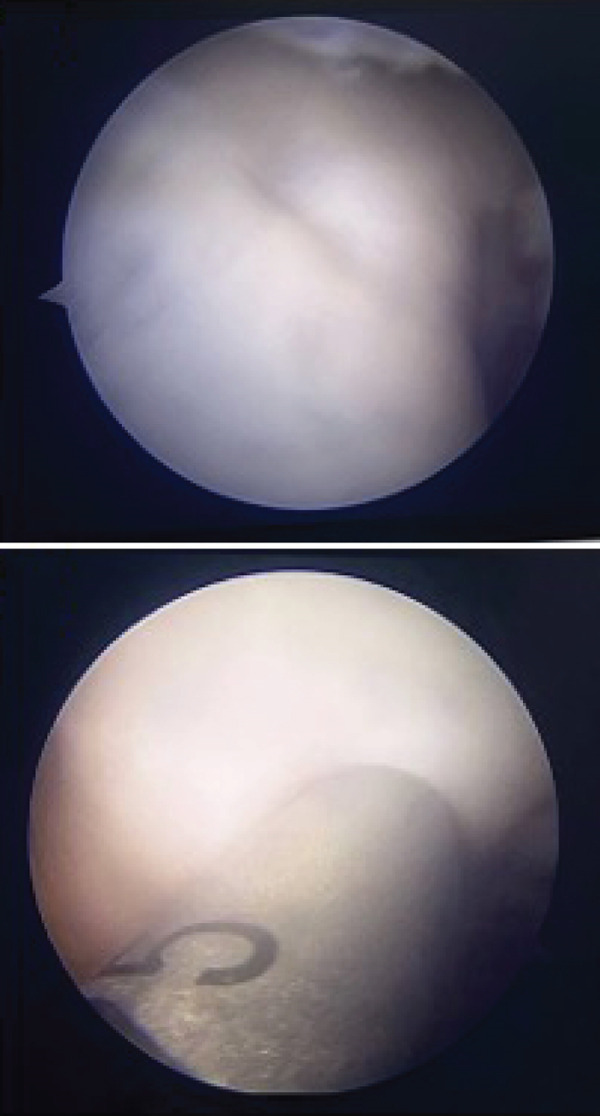
(a)

**Figure:**
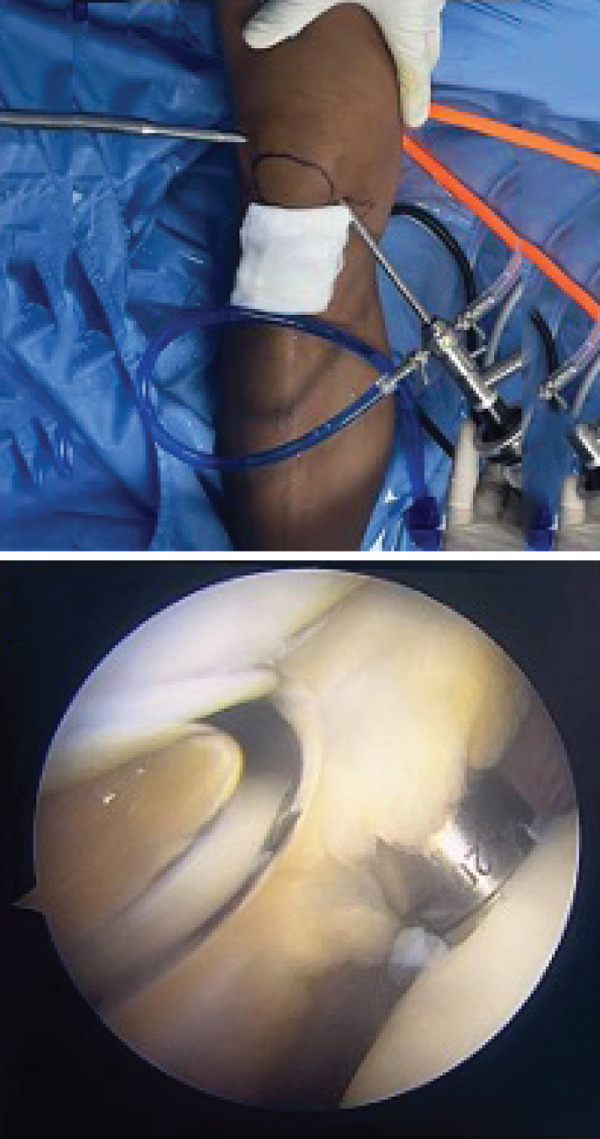
(b)

**Figure:**
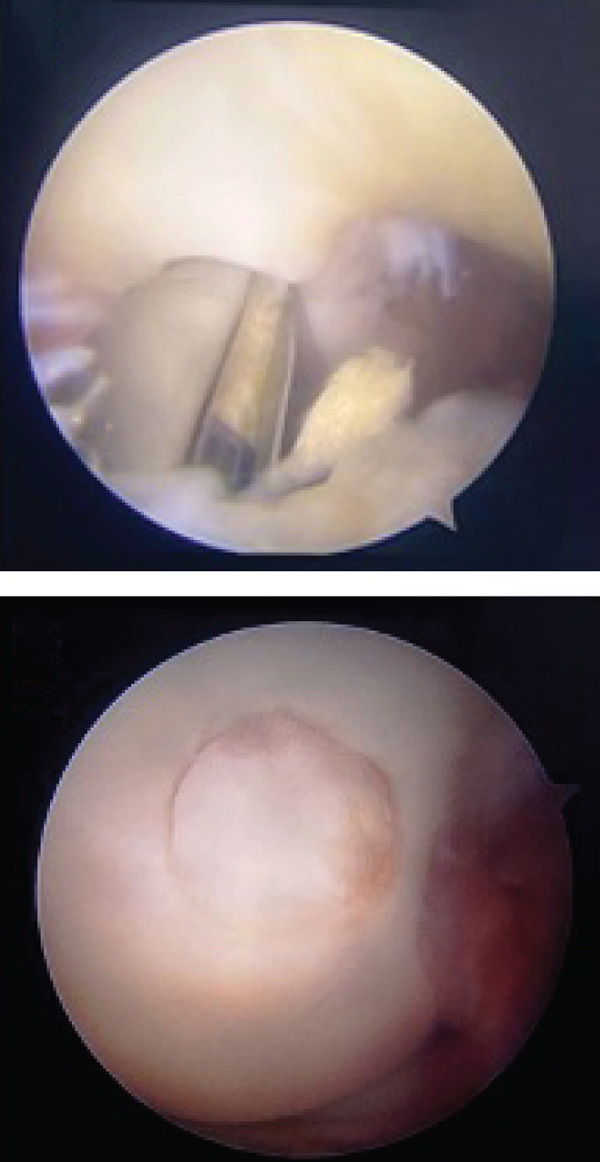
(c)

In the second patient, diagnostic arthroscopy revealed an intact ACL, PCL, medial and lateral meniscus, and intact medial and lateral gutters. However, we identified a focal OCD (Figure 6a) involving the medial femoral condyle, measuring approximately 1.8 cm in diameter. Additionally, thickening of the medial plica was noted. Similar to the previous case, stabilization of the donor region was done (Figure 6c,d). Osteochondral cylindrical plugs were harvested from the nonweight‐bearing region of the lateral femoral condyle, with diameters of 8, 6, and 6 mm (Figure 6b). The defect site was then reconstructed using an 8‐mm plug placed superiorly and two 6‐mm plugs placed inferiorly (Figure 6f). Upon evaluation through passive flexion and extension, the grafts in both cases were found to be well seated and stable within the defect site. The procedure concluded with layered closure of the portals, and the patient tolerated the procedure well under general anesthesia. Following donor implantation in both patients, evaluation of flexion and extension of the knee was carried out, and it was found that the graft was implanted securely.

Figure 6Mosaicplasty procedure of the second case. (a) Identification of osteochondral defect, revealing an 18‐mm defect, (b) harvesting of osteochondral plug from the lateral femoral condyle (nonweight‐bearing portion), (c, d) preparation of donor site, (e) applying the donor plug into the defect, it is then tamped with a tamper to achieve a leveled surface of the plugs and surrounding cartilage, and (f) final construct.

**Figure:**
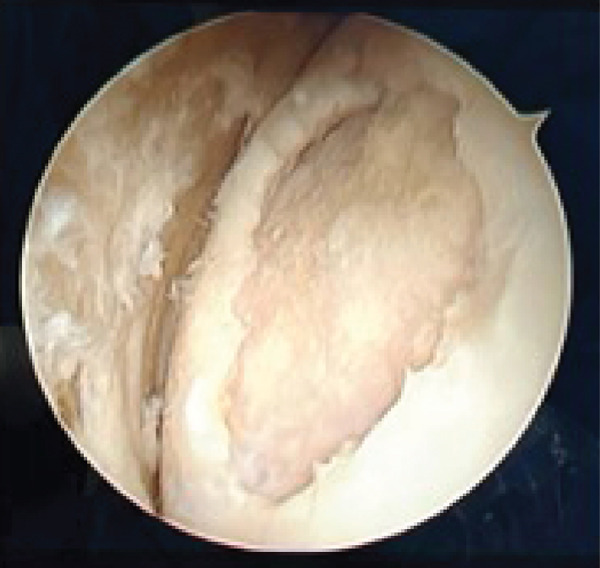
(a)

**Figure:**
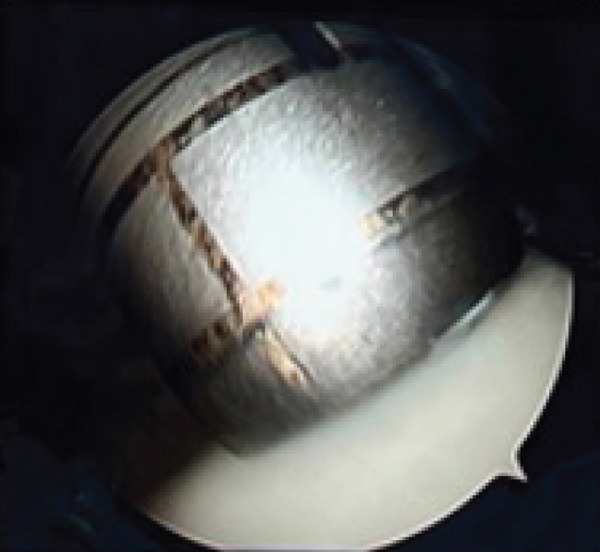
(b)

**Figure:**
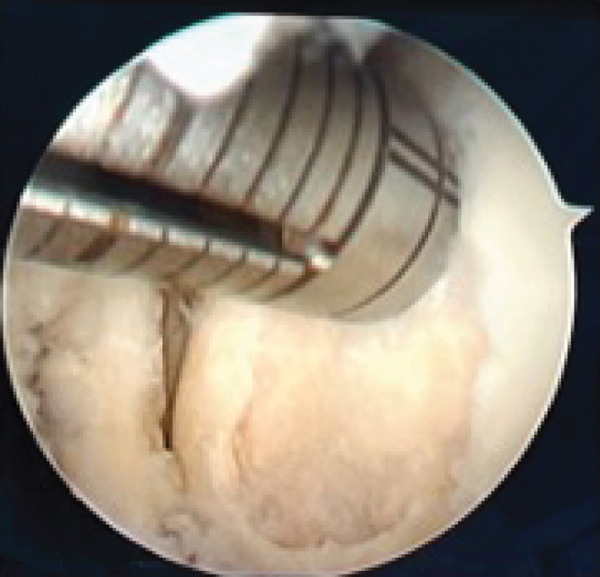
(c)

**Figure:**
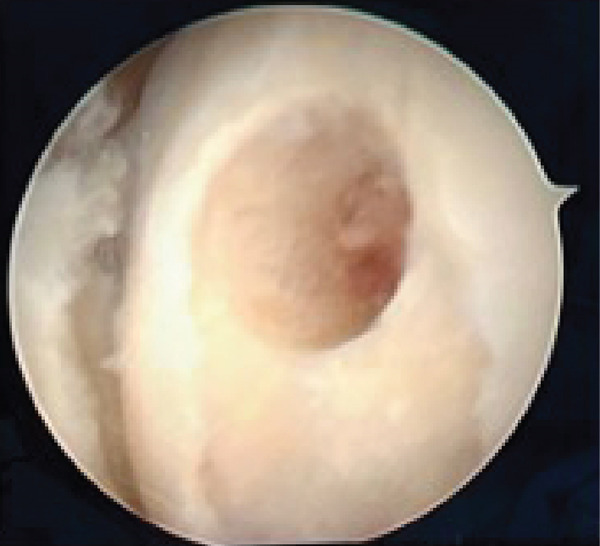
(d)

**Figure:**
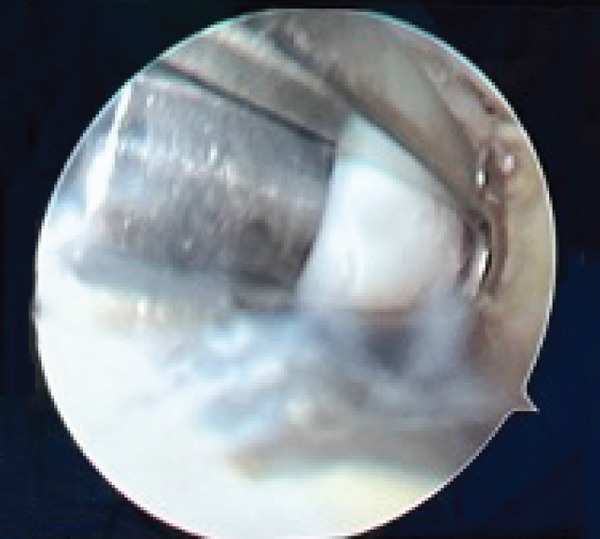
(e)

**Figure:**
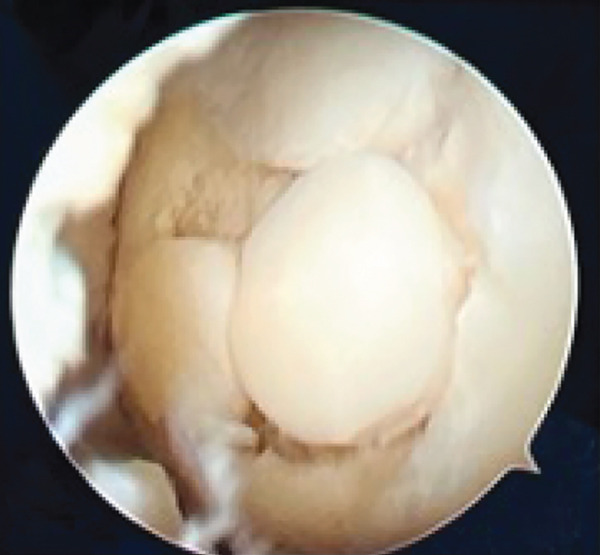
(f)

All surgical procedures were performed by the author (A.M.T.L), consultant in orthopedic sports surgery. Following surgery, patients were immobilized with a knee brace locked in extension, and prescribed a standardized rehabilitation program. Initial postoperative rehabilitation is aimed at regaining ROM while protecting the donor plugs. Patients were encouraged to gradually increase knee ROM to achieve 90° knee flexion by the second postoperative week. Afterwards, the brace was set in a flexible range so that patients could start passive and active ROM exercises. Knee ROM is gradually increased to reach 125° by the sixth postoperative week. Weight‐bearing was increased as tolerated. Around 7–12 weeks postsurgery, the focus of the rehabilitation program shifts to regaining movement control and strength, which will reduce stress to the articular surface. These included proprioceptive exercises, balance drills, and quadriceps strengthening exercises, which were gradually increased in intensity for 3 months. The final stage of rehabilitation is aimed at preparing the patient to return to sports (RTS) by regaining dexterity through low‐ to high‐velocity exercises that train movement control.

The preoperative and postoperative outcomes were assessed clinically and objectively. Two of the clinical assessments used were the Tegner–Lysholm Knee Scoring Scale and IKDC score (Table 2).

**Table 2:** Evaluation score in preoperative and postoperative arthroscopic mosaicplasty of the knee.

Scoring		Patient 1	Patient 2
IKDC	Preoperative	20.7	46.0
	6 month postoperative	60.2	57.5
	1 year postoperative	85.1	—
Tegner–Lysholm	Preoperative	21.0	61.0
	6 month postoperative	76.0	84.0
	1 year postoperative	95.0	—

Aside from scoring through IKDC and Tegner–Lysholm scale, another clinical assessment is the patient′s VAS pain level and knee ROM. At 1 year postoperative follow‐up of the first case, the patient demonstrated VAS pain levels of 0–1, and knee ROM of 0°–150°. In the second case, she achieved her first full weight‐bearing step 1 month postsurgery, bearing minimal pain (VAS 2–3), she then continued physiotherapy and reported continuous decrease in pain levels. At the 6 month follow‐up postsurgery, the patient′s VAS pain levels have achieved 0–1. Aside from pain, the patient′s knee ROM was also restored to 0°–150°, similar to the first case. The IKDC and Tegner‐Lysholm score for Patient 2 was unable to be assessed, as the patient is lost to follow‐up. Table 3 highlights the timeline in the management of both cases.

**Table 3:** Table encompassing both cases′ timeline.

Milestone	Patient 1	Patient 2
Onset of complaint	April 2022	June 2023
Additional consultation prior to first contact with the Orthopadic and Traumatology Department of Rumah Sakit Cipto Mangunkusumo (RSCM)	April 2022: Regular visits to bonesetter	July 2023: Bonesetter
	December 2022: First medical contact with local hospital	April 2023: First medical contact in a community health center
	January–March 2023: Physiotherapy	
First contact with the Orthopedics and Traumatology Department of RSCM	October 2023	May 2024
Diagnosis	November 2023	May 2024
Mosaicplasty surgery	December 2023	October 2024
Rehabilitation milestone(s)	Patient 1	Patient 2
Gradual increase active ROM until 90° flexed knee	1–2 weeks post‐op (December 2023)	1–2 weeks post‐op (October 2024)
Full range of motion	March 2024	November 2024
Absence of pain	March 2024	April 2025
Return to sports	October 2024	Patient does not return for control

## 3. Results and Discussion

Mosaicplasty was first introduced in the 1980s and utilizes autologous grafting from nonweight‐bearing donor sites, where one or more cylindrical plugs consisting of subchondral cartilage and bone are harvested and implanted into the defect. This technique has demonstrated high effectiveness and favorable long‐term outcomes for the management of OCDs within weight‐bearing joints, reaching 94% for the treatment of lesions of the femoral condyle. A substantial number of patients reported a reduced quality of life due to osteochondral damage of the knee. The goal in osteochondral lesion management is to preserve the original cartilage and bone. Options for treatment include nonsurgical management such as immobilization and weight‐bearing limitations, commonly indicated for cases of stable juvenile osteochondritis defects, which have a better overall prognosis [1]. A systematic review demonstrated that conservative treatments have an overall healing rate of 61.4% with large variability (10.4%–95.8%), and factors such as large lesion size, severe stages, and older age were associated with poor outcomes [2].

In adult patients, lesions rarely heal without surgical intervention, and such an approach is recommended for adult patients or juvenile cases nearing the end of the growth period that do not respond to a conservative approach. Surgical approach is aimed at restoring joint stability and preventing further damage. For smaller lesions less than 2 cm^2^, surgical options typically include subchondral drilling, microfracture, and fragment stabilization using an arthroscopic approach. Cases with larger lesions or loose bodies may require more extensive procedures such as autologous chondrocyte implantation or mosaicplasty [3].

Various studies had demonstrated the superiority of mosaicplasty in terms of RTS rate, where Minzlaff et al. reported that from 30 skeletally mature athletic patients, with a femoral condyle defect size of 2.05 ± 0.88 cm^2^, the RTS rate to prior level of sport was 76.7% [12]. Gudas et al. reported from a cohort of 60 athletes, RTS rate reached 100% of the entire cohort, with 86% of those that received mosaicplasty for repair of OCD returned to prior level of sports with a mean time of 6.8 months postoperatively [13].

From our experience, both patients had an OCD of the femoral condyle confirmed by diagnostic arthroscopy. Patient 1 was a 17‐year‐old male, skeletally immature, and engaged in a high level of physical activities; whereas Patient 2 was a skeletally mature, 28‐year‐old female. Our study demonstrated that both patients had significant improvement in knee function and pain following mosaicplasty. There was an improvement of 74 and 23 points in Tegner–Lysholm knee score and 64.4% and 11.5% in IKDC score for Patients 1 and 2, respectively, after the longest follow‐up period for each patient. We found that Patient 1 achieved full ROM, absence of pain, and restored sense of stability of the joint within 3 months postoperatively. The patient returned to sports within 8 months following the operation. Patient 2 achieved a ROM of > 150° and was able to do her first step 1 month post‐op with minimal pain, with the pain fully absent at 6 months follow‐up after a series of routine physiotherapy.

The rehabilitation protocol following mosaicplasty is generally divided into three phases, the initial phase focusing on regaining ROM (0–6 weeks), strengthening and regaining movement control (7–12 weeks), and preparation for RTS (≥ 16 weeks). During the initial phase, patients are encouraged to gradually increase knee ROM, patellar mobility, and restore leg control while protecting the postsurgical knee. This is achieved through ROM exercises such as passive knee flexion and full knee extension. The usage of the continuous passive motion (CPM) machine has been shown to supplement the initial stage of recovery by promoting movement of nutrients within the synovial fluid into the donor cartilage and stimulating an anabolic response within the chondrocytes [14]. During the second phase, the focus of the program shifts to regaining good control of gait and functional movements, which is achieved through exercises such as quadriceps strengthening, proprioceptive drills, and gait drills. The final phase of rehabilitation involves reintroducing patients to sport and specific movements, including those that introduce impact to the postsurgical knee. This stage of rehabilitation involves drills such as lunges, squats, single‐leg balance and proprioceptive drills, and impact control exercises. Eight months postsurgery, patients can be reintroduced to moderate impact activities such as jogging, and by 10 months, to high‐impact sports such as basketball and soccer [15].

Our study demonstrated a significant improvement in the patient′s knee function and pain within 6 months and 1 year following mosaicplasty treatment (Table 2). A similar study reported that in a 22‐year‐old female with OCD in both the lateral and medial femoral condyles of both knees, knee function measured by IKDC score improved from 38 preoperatively to 85 at 1 year following mosaicplasty [16]. Furthermore, another recently published report highlighted that autograft mosaicplasty produced resolution of pain and restoration of ROM within 10 days following the procedure, and patient was able to participate in joint‐friendly activities such as cycling within the first 2.5 months, and eventually running at 4 months postoperatively [17]. Both studies were carried out using an open approach, and in contrast, our study adds to the current literature by demonstrating that the procedure can be carried out arthroscopically and produced similar favorable outcomes, and is associated with lower risks of complications such as hemarthrosis.

There are several techniques in the treatment of OCDs of the knee, of which the gold standard remains a debatable topic. Mosaicplasty has been associated with benefits such as complete removal of necrotic subchondral tissue within the defect, grafts hyaline cartilage with superior mechanical properties compared with fibrocartilage, addresses both articular cartilage defects as well as subchondral defects, and being a single‐step procedure that can be carried out arthroscopically with satisfactory long‐term success rates [18, 19]. On the other hand, it is associated with disadvantages such as potential donor‐site morbidity, size mismatches, and limited availability of areas suitable for graft harvesting [19]. As such, mosaicplasty is commonly preferred for cases with defect sizes > 2 cm^2^, whereas smaller lesions are offered subchondral drilling with possible debridement and fragment stabilization. However, our experience demonstrated that for cases with defects smaller than 2 cm^2^ such as the case of Patient 2, mosaicplasty is proven to be a highly effective intervention that produces an improvement in IKDC score of 64.4% and Tegner–Lysholm score of 74 points, and allowed the patient to RTS as early as 9 months following mosaicplasty, and absence of pain at the end of the follow‐up. The clinical trial published by Bentley et al. reported that for smaller lesions (< 2 cm^2^), mosaicplasty is found to be as effective as ACI, introducing cheaper, more accessible treatment as an alternative option for recalcitrant lesions [20].

The effectiveness of mosaicplasty has been reported worldwide, with studies even reporting long‐term benefits. That being said, the adoption and availability of this particular method remain limited across different regions of the world. Specifically in Indonesia, cartilage restoration procedures such as mosaicplasty are still emerging, with limited published data in regard to their outcomes, feasibility, and perioperative considerations [21]. To our knowledge, this case series represents one of the earliest reported experiences of arthroscopic‐assisted mosaicplasty in Indonesia, thereby providing region‐specific clinical evidence in support of future implementation. This is particularly relevant in a setting with limited resources or access to cell‐based cartilage repair techniques such as ACI and MCI.

Despite its promising results, this case series has several limitations worth acknowledging. First, with this study being a case series, inherently it will not provide a causal conclusion in regards to the effectiveness of mosaicplasty for OCDs. Furthermore, both cases presented are particularly heterogeneous, representing different age groups, skeletal maturity, defect location, and size, which limits meaningful comparison of the two cases and only offers a showcase‐like report. Adding on to the heterogeneity, both cases generally have long‐awaited time from incident to first medical contact, and both seek other means prior to medical contact such as bonesetter and physiotherapy. This should be acknowledged as it may introduce an additional variable that affects the baseline cartilage condition and therefore influences outcomes, given that preoperative symptom duration has been identified as an independent variable associated with reduced RTS rates following cartilage repair, as highlighted by Mithoefer et al. [22] Notably, however, delayed presentation in this patient population is not uncommon, with mean symptom duration before surgery reported to exceed 5 years [23]. The last limitation is there is no systematic monitoring in regards to the donor‐site morbidity, so there is no long‐term documentation of consequences for the donor‐site harvesting.

This case series, acknowledging its inherent limitations, serves as an early clinical documentation of arthroscopic‐assisted mosaicplasty performed in an Indonesian clinical setting. Despite the absence of a significant analysis and conclusion, this study can serve as a reference to orthopedic surgeons who are facing a similar scenario, widening their options. It is with the authors′ hope that this case series can contribute to the limited local evidence and also fuel future research to support adoption of this procedure broadly.

## 4. Conclusion

In conclusion, our study recommends the use of arthroscopic mosaicplasty for cases of OCDs of the femoral condyle. It is advantageous as it is a single‐step procedure that can be carried out arthroscopically, utilizes mechanically superior autologous hyaline graft, and addresses both articular cartilage and subchondral bone defects. Our study also demonstrated that it is applicable for smaller lesions, produces excellent knee functional score at 1 year follow‐up, while minimizing risks of subchondral bone necrosis associated with subchondral drilling and microfracture. However, this study has several limitations that need to be acknowledged, starting with the heterogeneity of the two cases, no causal analysis, long duration of symptoms prior to first medical contact, and no documentation of donor‐site morbidity.

## Funding

No funding was received for this manuscript.

## Ethics Statement

This case report is exempted from ethical approval by the IRB as the published data are anonymized.

## Consent

The authors declared that the subjects of this study have given consent to be included in the study. Written consent for publication was obtained from both patients, and parental consent was obtained for the subject of Case 1. All data (e.g., clinical data and radiologic imaging) do not contain identifying information.

## Conflicts of Interest

The authors declare no conflicts of interest.

## Supporting information


**Supporting Information** Additional supporting information can be found online in the Supporting Information section. CARE checklist.

## Data Availability

The data that support the findings of this study are available on request from the corresponding author. The data are not publicly available due to privacy or ethical restrictions.
