# Supplementary material for: Arthroscopic‐Assisted Mosaicplasty for the Repair of International Cartilage Repair Society (ICRS) Grading System Grade 4 Osteochondral Defect: A Case Report of Two Patients
Source: Case Rep Orthop. 2026 Apr 6;2026:1667776. doi: 10.1155/cro/1667776 (PMC13051788; doi:10.1155/cro/1667776)
Supplement: Supplementary file 1 — Supporting Information Additional supporting information can be found online in the Supporting Information section. CARE checklist. [file CRO-2026-1667776-s001.pdf]

CARE case report guidelines

CARE Checklist of information to include when writing a case report

(cc) BY-NC-ND

Topic	Item	Checklist item description	Reported on Line
Title	1	The diagnosis or intervention of primary focus followed by the words "case report"	1 - 4
Key Words	2	2 to 5 key words that identify diagnoses or interventions in this case report, including "case report"	62 - 63
Abstract (no references)	3a	Introduction: What is unique about this case and what does it add to the scientific literature?	31 - 36
	3b	Main symptoms and/or important clinical findings	38
	3c	The main diagnoses, therapeutic interventions, and outcomes	38 - 45
	3d	Conclusion—What is the main "take-away" lesson(s) from this case?	56 - 69
Introduction	4	One or two paragraphs summarizing why this case is unique (**may include references**)	70 - 72
Patient Information	5a	De-identified patient specific information.	128; 153
	5b	Primary concerns and symptoms of the patient.	128 - 131; 153 - 156
	5c	Medical, family, and psycho-social history including relevant genetic information	128 - 133; 153 - 157
	5d	Relevant past interventions with outcomes	131 - 133; 156 - 157
Clinical Findings	6	Describe significant physical examination (PE) and important clinical findings.	135 - 137; 157 -159
Timeline	7	Historical and current information from this episode of care organized as a timeline	261
Diagnostic Assessment	8a	Diagnostic testing (such as PE, laboratory testing, imaging, surveys).	137 - 143; 157 - 165
	8b	Diagnostic challenges (such as access to testing, financial, or cultural)	-
	8c	Diagnosis (including other diagnoses considered)	142 - 143; 163 - 165
	8d	Prognosis (such as staging in oncology) where applicable	-
Therapeutic Intervention	9a	Types of therapeutic intervention (such as pharmacologic, surgical, preventive, self-care)	182 - 184
	9b	Administration of therapeutic intervention (such as dosage, strength, duration)	185 - 188
	9c	Changes in therapeutic intervention (with rationale)	186 - 188
Follow-up and Outcomes	10a	Clinician and patient-assessed outcomes (if available)	246 - 260
	10b	Important follow-up diagnostic and other test results	246 - 260
	10c	Intervention adherence and tolerability (How was this assessed?)	-
	10d	Adverse and unanticipated events	-
Discussion	11a	A scientific discussion of the strengths AND limitations associated with this case report	-
	11b	Discussion of the relevant medical literature **with references**.	267 - 279; 281 - 287; 290 - 296; 311 - 328; 330 - 341; 343 - 360; 373 - 389
	11c	The scientific rationale for any conclusions (including assessment of possible causes)	267 - 269; 344 - 349
	11d	The primary "take-away" lessons of this case report (without references) in a one paragraph conclusion	392- 397
Patient Perspective	12	The patient should share their perspective in one to two paragraphs on the treatment(s) they received	-
Informed Consent	13	Did the patient give informed consent? Please provide if requested	Yes ☑ No ☐
